# Supplementary material for: Synthetic molecular evolution of hybrid cell penetrating peptides
Source: Nat Commun. 2018 Jul 2;9:2568. doi: 10.1038/s41467-018-04874-6 (PMC6028423; doi:10.1038/s41467-018-04874-6)
Supplement: Supplementary file 1 — Supplementary Information [file 41467_2018_4874_MOESM1_ESM.pdf]

Supplementary Material

Synthetic molecular evolution of hybrid cell penetrating peptides.

Authors: W. Berkeley Kauffman, Shantanu Guha, and William C. Wimley*

Affiliations:

Department of Biochemistry and Molecular Biology, Tulane University School of Medicine, 1430 Tulane Avenue, New Orleans, LA 70112

*To whom correspondence should be addressed: wwimley@tulane.edu

Supplementary Figure 1.

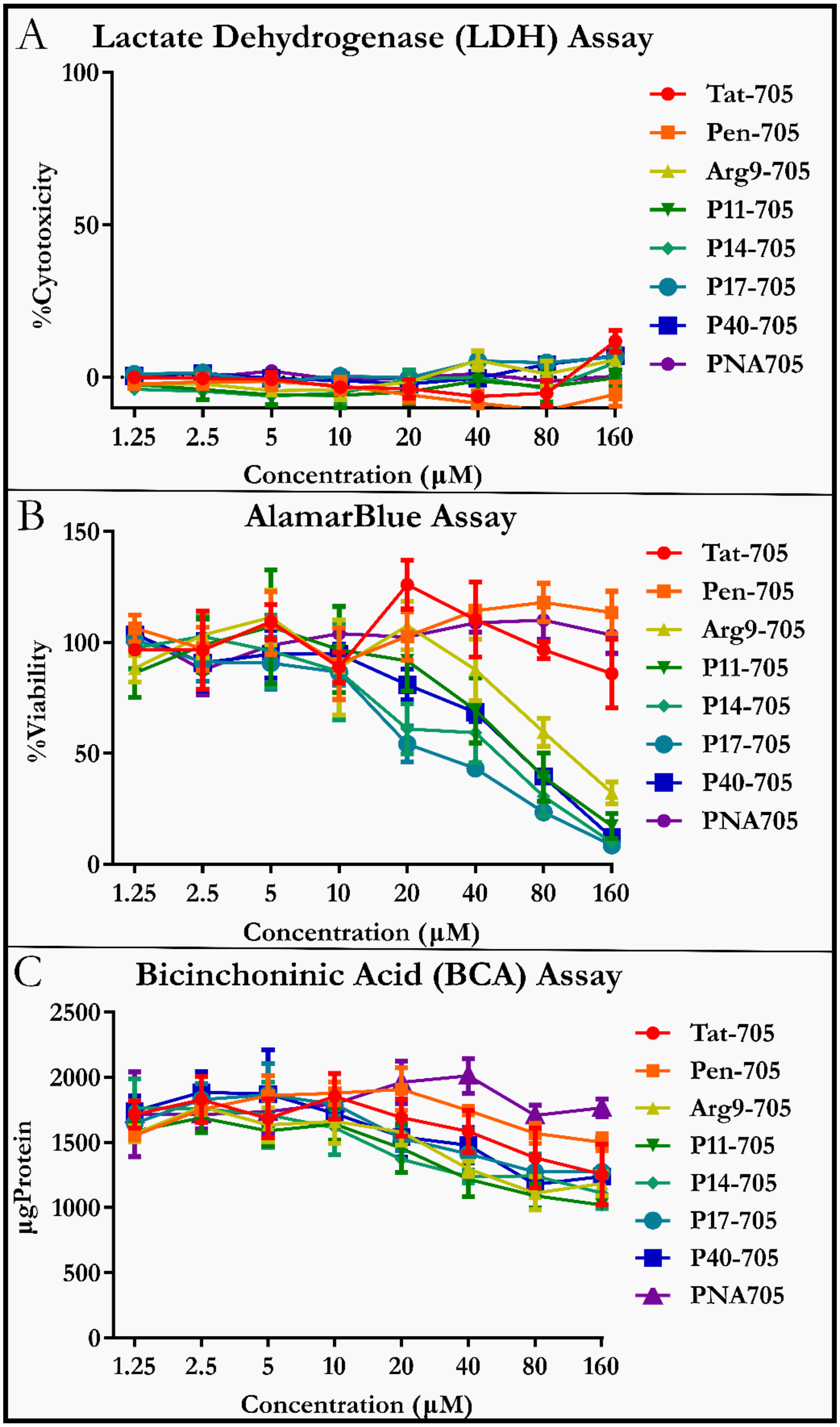

Supplementary Figure 1: Cell viability assays for peptide-PNA chimeras. HeLa cells were treated with treated with varying concentrations of peptide up to 160 μM and were analyzed using A) lactase dehydrogenase (LDH) assay B) alamarBlue cell viability reagent, and C) bicinchoninic acid (BCA) assay. The LDH assay is a measure of acute cytotoxicity and detects LDH released by cells because of membrane damage. Metabolically active cells maintain an internal reducing environment and convert the alamarBlue reagent into a fluorescent molecule resorufin at a rate that is proportional to both the health of the cell and the number of cells in the well. The BCA assays the total protein and is a proxy for cytotoxicity as cells exposed to toxic peptides do not efficiently translate protein. The LDH assay was performed immediately following exposure to the peptide construct and is a measure of acute cytotoxicity whereas the alamarBlue and BCA assays were performed after 24 hours and would reflect latent cytotoxic effects. N=4 for all experiments.

Supplementary Figure 2.

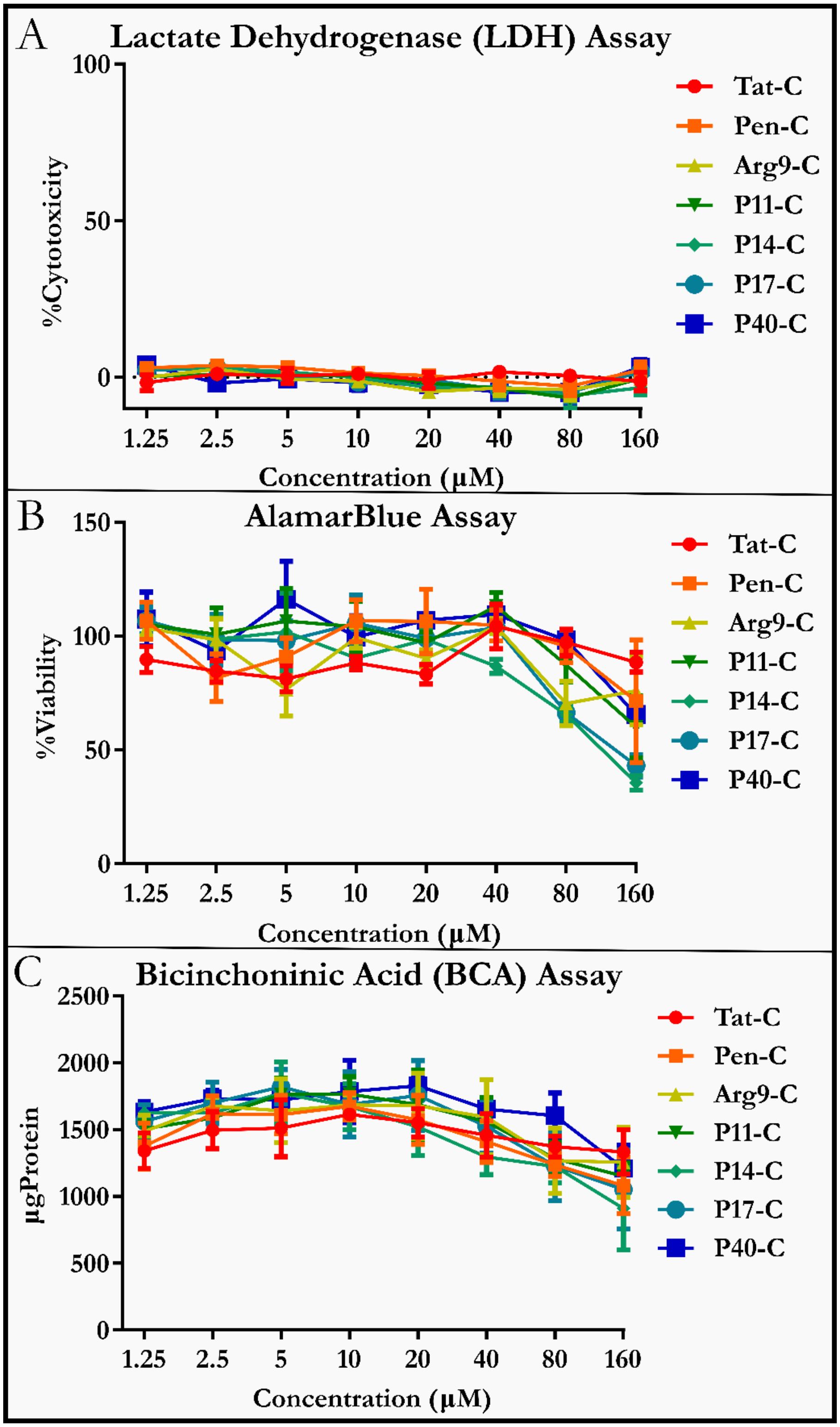

Supplementary Figure 2: Cell viability assays for peptides with C-terminal cysteine. HeLa cells were treated with treated with varying concentrations of peptide up to 160 μM and were analyzed using A) lactase dehydrogenase (LDH) assay B) alamarBlue cell viability reagent, and C) bicinchoninic acid (BCA) assay. The LDH assay is a measure of acute cytotoxicity and detects LDH released by cells because of membrane damage. Metabolically active cells maintain an internal reducing environment and convert the alamarBlue reagent into a fluorescent molecule resorufin at a rate that is proportional to both the health of the cell and the number of cells in the well. The BCA assays the total protein and is a proxy for cytotoxicity as cells exposed to toxic peptides do not efficiently translate protein. The LDH assay was performed immediately following exposure to the peptide construct and is a measure of acute cytotoxicity whereas the alamarBlue and BCA assays were performed after 24 hours and would reflect latent cytotoxic effects. N=4 for all experiments.

Supplementary Figure 3.

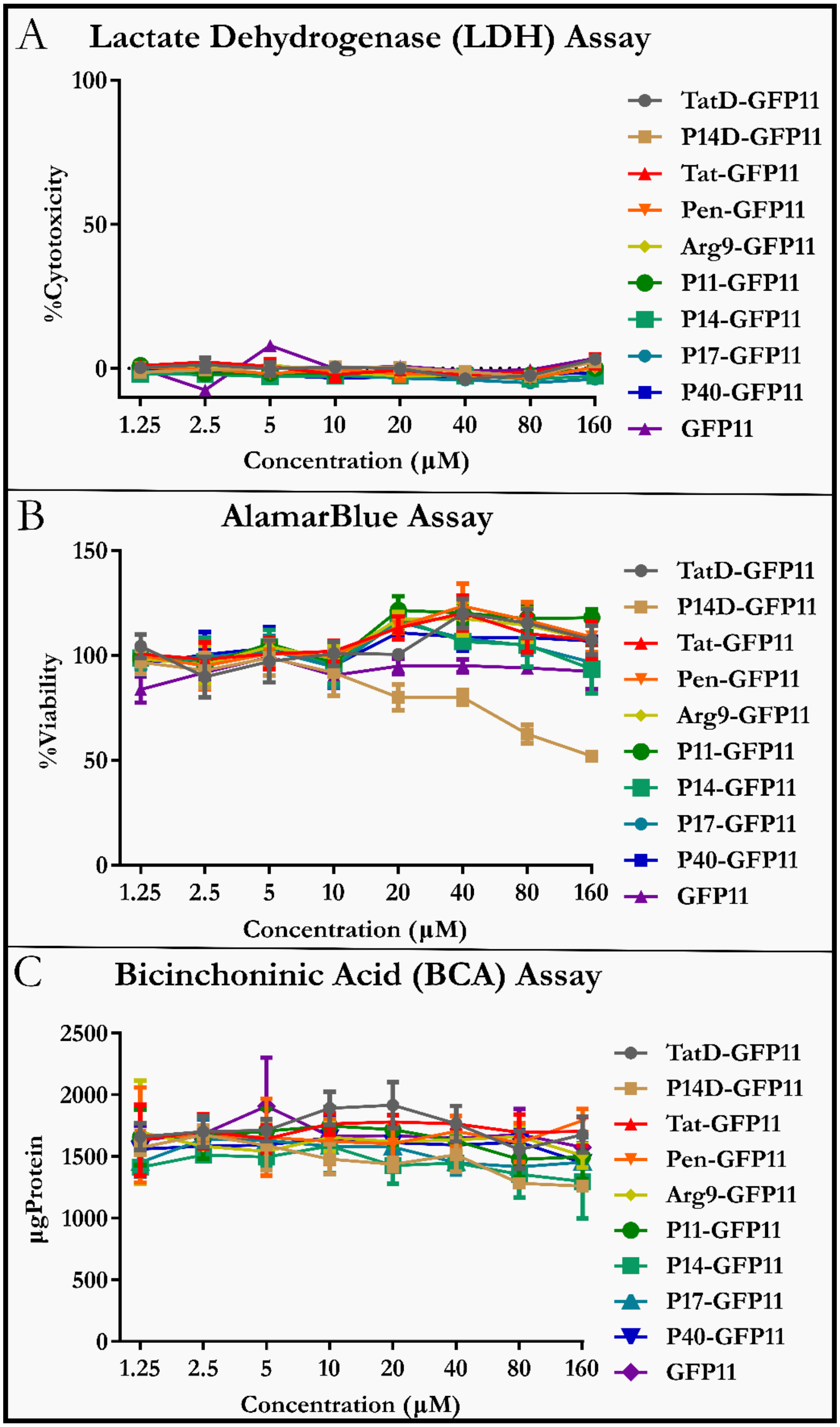

Supplementary Figure 3: Cell viability assays for peptide-GFP11 constructs. HeLa cells were treated with treated with varying concentrations of peptide up to 160 μM and were analyzed using A) lactase dehydrogenase (LDH) assay B) alamarBlue cell viability reagent, and C) bicinchoninic acid (BCA) assay. The LDH assay is a measure of acute cytotoxicity and detects LDH released by cells because of membrane damage. Metabolically active cells maintain an internal reducing environment and convert the alamarBlue reagent into a fluorescent molecule resorufin at a rate that is proportional to both the health of the cell and the number of cells in the well. The BCA assays the total protein and is a proxy for cytotoxicity as cells exposed to toxic peptides do not efficiently translate protein. The LDH assay was performed immediately following exposure to the peptide construct and is a measure of acute cytotoxicity whereas the alamarBlue and BCA assays were performed after 24 hours and would reflect latent cytotoxic effects. N=4 for all experiments.

Supplementary Figure 4.

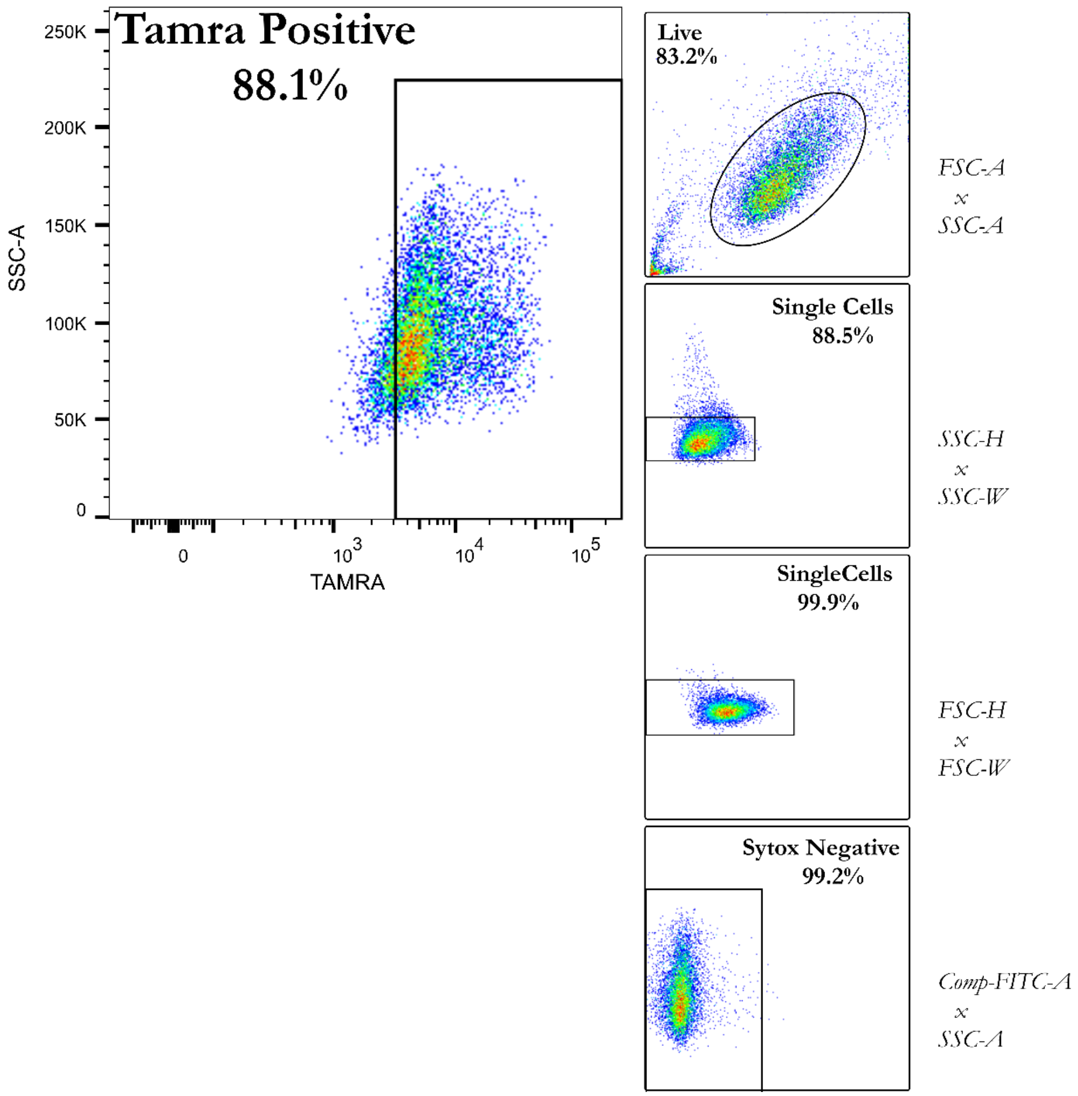

Supplementary Figure 4. Flow cytometry gating strategy for Peptide-C-TA treated cells. In this example, HeLa cells were treated with 5 μM P17-C-TA for 30 minutes at 37°C. Live cells were identified by forward and side scatter. Doublets were excluded in two gating steps using SSC-H x SSC-W and FSC-H x FSC-W. Cells with compromised membranes permitting SYTOX green entry were excluded. The percent of the remaining cells testing positive for TAMRA fluorescence were defined as those with a fluorescence value ≥3000. Additionally, the mean fluorescence for all SYTOX negative cells was recorded.

Supplementary Figure 5.

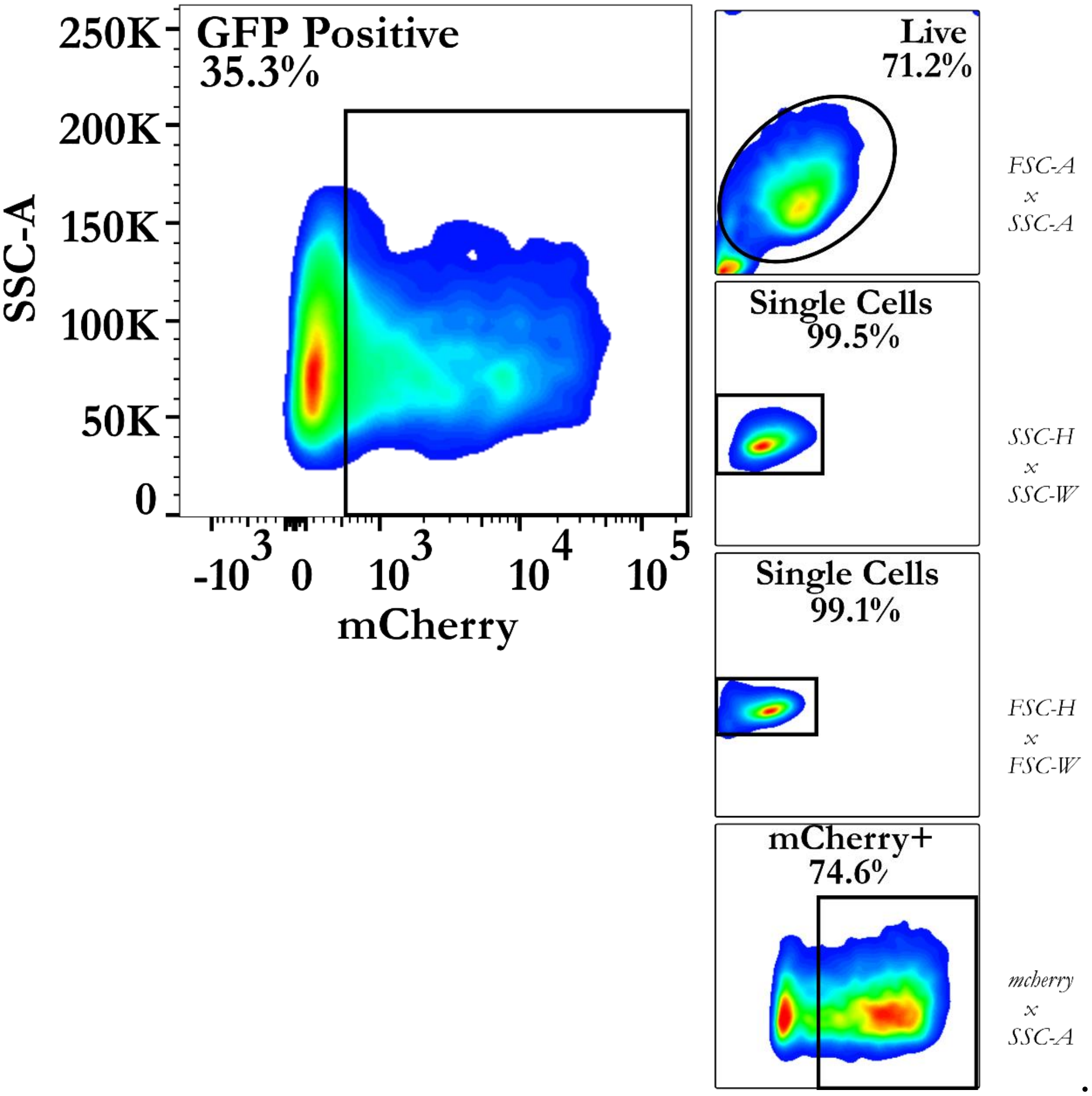

Supplementary Figure 5: Flow cytometry gating strategy for PDEP—GFP11 treated cells. Here, HeLa p_mCherry-GFP1-10 cells were treated with 10-40 μM P17-C-TA for 30 minutes at 21°C or 37°C. Live cells were identified by forward and side scatter. Doublets were excluded in two gating steps using SSC-H x SSC-W and FSC-H x FSC-W. Cells not expressing the mCherry: GFP1-10 fusion protein were excluded based on mCherry fluorescence. The percent of the mCherry positive cells testing positive for GFP fluorescence were defined as those with a fluorescence value ≥500 which was the maximum GFP fluorescence of untreated cells. Additionally, the mean GFP fluorescence for all mCherry positive cells was recorded.
